# FAIR, open, and free does not mean no restrictions

**DOI:** 10.1016/j.patter.2021.100339

**Published:** 2021-09-10

**Authors:** Keith G. Jeffery

**Affiliations:** 1 British Geological Survey, Keyworth NG12 5GG, UK

## Abstract

Commonly, these terms are used incorrectly, causing much confusion and misunderstanding. Actually, no digital asset is completely FAIR, open, and free, and most would be classified somewhere on a spectrum of FAIRness, openness, and freedom from cost. Key restrictions hindering the ideal include privacy (personal data protection), licensing, access/use authorization, security, and costs that may be absorbed by an organization.

## Main text

### Proposition

“FAIR,” “open,” and “free” are rarely used correctly to describe access to digital assets^1^ such as (web)services, datasets, data products, software code, software binaries or libraries, workflows, or publications (white and gray). In fact, digital assets—expected to be or described as FAIR, open, and free—are subject to many restrictions. Thus, it is difficult to state whether an asset is FAIR, or open, or free because usually it is not a binary valued characteristic—a spectrum of possibilities exists—for each of FAIR, open, free. Depending on the restrictions applied to a particular digital asset, the end user will need to decide whether the digital asset is sufficiently FAIR, open, and free for their purpose at that time.

### FAIR

Access to a digital asset is FAIR if the asset conforms to the FAIR principles (findability, accessibility, interoperability, reusability).^2^ However, it is generally accepted that there are no absolute measures of FAIRness; in fact, FAIRness has been described as a journey toward enabling access. Excellent work by the Research Data Alliance (RDA) FAIR Data Maturity Model Working Group led to a model^3^ used widely, and adopted by other FAIR initiatives, to assess FAIRness. Many existing metadata standards, and associated software processes, do not support fully FAIR access.

### Open

Access to a digital asset is open if there are no barriers to its use.^4^ Most digital assets have a license that, depending on the license type, is likely to restrict access or impose conditions on the licensee—minimally, acknowledgment of the licensor—but may also include restrictions on licensing of digital assets derived from the original asset. Licenses such as Creative Commons^5^ CC-BY-NC prohibit commercial use.

### Free

Pedantically, no digital asset is free to access, since energy, which has a cost, is used. However, generally “free” with respect to digital assets means “toll-free”; i.e., no charge is made for access. In the research publications world, both green and gold open access is free to the reader, but in the case of gold, an author has paid for the publication. In the green open access world, there are costs (time/effort) associated with the author depositing the publication in a repository as well as the infrastructure costs of maintaining the repository. Similarly, other digital assets (such as datasets) associated with research publications may not be free.

### Situation

In practice, a digital asset is usually considered to be FAIR, open, and free if (1) it is easily accessible using metadata, (2) any license allows unrestricted use except for acknowledgment (which is correct professional practice anyway), and (3) there is no charge for access. However, (1) the digital asset may include personal data, especially in the metadata, which means that the person owning the personal data has to have granted permission for its use either implicitly (legitimate business use) or explicitly under the General Data Protection Regulation (GDPR) in Europe^6^ and similar legislation elsewhere; (2) users of digital assets should also be aware that it is likely that their identity (personal data) is recorded for purposes of tracking usage, managing access rights, and potentially bettering the user experience by improving query precision and accuracy and recommending related digital assets of interest.

As well as licenses, digital assets may also be protected by organizational policies controlling access to them. Users usually have roles (in which they act), and associated with those roles are permissions to access digital assets, possibly with a temporal duration. A digital asset may be declared as available to anyone (person or software agent acting for the person) in any role for any purpose, at any time, without cost. This is the liberal end of the open and free spectrum; whether it is FAIR depends largely on the quantity and quality of metadata related to the digital asset. At the other end of the spectrum, a digital asset may be available only to a selected registered user in one particular role for a single purpose within a temporal duration, for a fee. This is clearly neither open nor free. There are many possible states between these two extremes (Table 1).

**Table 1:** Ends of the spectrum from liberal to restrictive

	Liberal	Restrictive
**Person**	any	single, identified
**Role**	any	single, identified
**Purpose**	any	single, identified
**Time**	any	single temporal duration
**Fee**	none	fee charged

### Restrictions

The restrictions that determine the extent to which an asset is available FAIR, open, and free are now covered.

Security and associated availability protect the digital asset from unavailability and any process causing corruption. Security prevents general unauthorized access that might cause corruption of the digital asset or disruption of services providing access, e.g., a distributed denial-of-service attack. Availability ensures that the digital asset is accessible, subject to any other restrictions (such as authorization). Availability is related to curation, likely defined by a digital asset management policy, including back-up copies and/or replicates for restoration of availability. Distributed and fragmented assets may improve both security and availability. Security breaches may be criminal. Security policies implemented in IT systems may preclude general access for particular users.

Privacy protects any personal data within or about the digital asset (e.g., in metadata) or personal data concerning access to the digital asset. The GDPR mentioned above is highly relevant here, and severe punishments are available. Personal data are common in metadata; examples are the owner of a digital asset, the manager of a digital asset. Some dataset formats include such data. Personal data within datasets in certain domains such as healthcare require particular protection (usually by authorization and anonymization). Similarly, personal data about the user/accessor of the digital asset may be recorded.

Rights and licenses protect a digital asset. Rights on the digital asset may be claimed (such as copyright or database right or even patenting) by the owner, and also licensing (by the licensor) may be more or less restrictive. Rights usually preclude use except under contractual conditions, which may include a fee. Licensing is a complex subject in its own right. It is common practice in research to use licenses that do not charge a fee and allow open use subject to acknowledgment. However, licensing may be different for different kinds of digital assets. As just one example, licensing a document may allow any derivative to be licensed under any other license (subject to acknowledgment), whereas licensing a piece of software may come with the restriction that derivatives must be under the same license (to preserve the openness under which the original software was licensed).

Authorization usually lies within an authentication, authorization, accounting infrastructure (AAAI) and is the relationship between the rights of the user to access a digital asset and the rights of the digital asset to be protected from access, usually defined by a combination of license and policies of an organization. Authorization of authenticated user access in a given user role (owner, manager, etc.) to a certain asset, in appropriate modalities (read, update, etc.), possibly within a certain time period, and subject to digital asset licensing, only, is permitted. Of course, the authorization could be for any user, in any role, utilizing any modality, upon any digital asset (with an open, free license) to give maximum open access.

Terms and conditions relate to the system controlling the digital assets that may have associated terms and conditions of use including, but not restricted to, disclaiming liability, agreeing to certain modalities of user behavior, and use of cookies.

### The key is metadata

Metadata describing each digital asset provide a way for the owner to control access to—and utilization of—the digital asset. Metadata describing the digital asset, ideally using terminology from a known and well-used vocabulary, will improve discovery of the digital asset. Richer metadata, providing, for example, details of the experimental method, the equipment used, the precision and accuracy of measurements, and calibration information, assists in contextualization, the assessment of the relevance and quality of the digital asset for the user’s purpose. Metadata covering the license (for information) and the parameters of the license (as attribute values or in an appropriate language) that control digital asset usage in an IT system through authenticated user authorization enable appropriate policies to be enforced, which may preclude use of a digital asset.

Metadata also cover curation, provenance, citation, and usage. Curation information may include not only where a digital asset is located but also whether it is fragmented into several locations and where versions are stored (e.g., for availability). Here curation intersects with provenance, which records everything that happens to a digital asset and, in so doing, everything a given person (or software agent acting on behalf of that person) has done across all digital assets. This is used (1) for reproducibility of research and (2) for audit purposes. These metadata records also provide a basis for recording citation of one digital asset by another, e.g., a dataset by a publication. Finally, they provide the basis for analyzing usage of digital assets, which may be necessary for recovering any fees due or to report to funding agencies, as well as input to analyses of performance leading to improvements.

### EPOS experience

In the EPOS research infrastructure,^7^ we are drawing together all these aspects into an integrated policy-driven set of mechanisms in the system including rich metadata, policy and license documents, guidelines, and consent at the user interface relying on an AAAI system based on the recommendations of AARC.^8^ This is very much a work in progress, since we also have to relate, for interoperability, to other research infrastructures and clusters of them and to initiatives such as the European Open Science Cloud (EOSC).^9^

**Figure 1:**
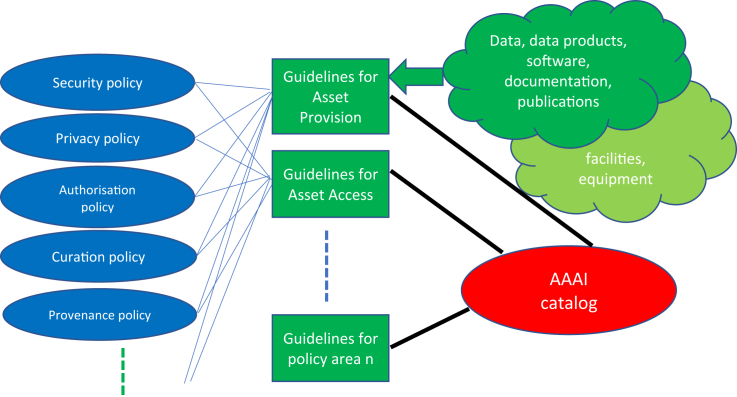
Policies, guidelines, and digital assets

First, the areas where policy is needed, relating to the IT system and management of digital assets, were defined. These areas are asset provision, asset access, personal data protection (including GDPR), security (including privacy and authorization), and responsible research and innovation (RRI), which covers the rights of the person. Second, the digital assets were defined based on discussions with providers and users. Third, the areas where guidelines were needed were defined, covering the policy areas and based on which policies related to which digital assets (Figure 1).

**Figure 2:**
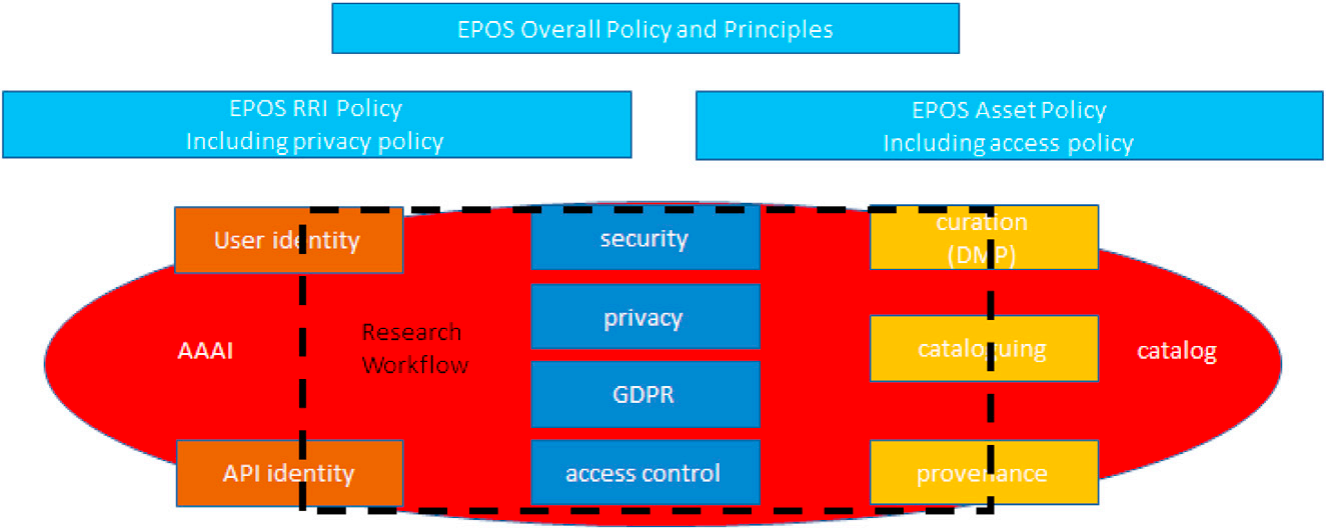
Policies implemented in IT

The way in which the guidelines, implementing the policies, are supported by the IT is illustrated in Figure 2.

From this, it is clear that system components for curation, cataloging, and provenance implement the guidelines (based on policies) relating to the digital assets. Similarly, system components for user identity—and any associated application processing interface (API) identity for software agents acting on behalf of the user—relate to RRI. In the intersection between the rights and responsibilities of assets, and those of users, lie system components for security, privacy, GDPR, and access control. A research workflow utilizes all of these components as indicated by the dashed rectangle. Finally, the system components mentioned rely on the system metadata catalog, which holds metadata about all assets and users, and the AAAI system, which controls the relationship between them.
